# Medical Wards of the Mercy Hospital

**Published:** 1873-12-01

**Authors:** N. S. Davis


### MEDICAL WARDS OF THE MERCY HOSPITAL.

SERVICE OF PROF. N. S. DAVIS, NOV. 6, 1873.

*Double Pneumonia.*—Mr. G——, a laboring man, aged about 30 years, was admitted into the hospital November 3d, 1873. He informed the house physician that he had been attacked only three days previously with headache, pains in the back and limbs, followed by fever, some cough, with soreness in the chest, and a loose condition of the bowels. At the time of his admission, his expression was dull, with slight flush on the cheeks; tongue slightly coated; skin but little above the natural temperature; respiration short and quick; pulse 100, soft and weak; bowels moving at the rate of eight or ten times in the twenty-four hours; abdomen moderately full and tympanitic; expectoration scanty, and tinged with blood, though the cough was not severe. He was directed a teaspoonful of the emulsion of turpentine and laudanum every three hours, and wheat-flour and milk-porridge for nourishment.

The same evening, however, he became extremely depressed; his skin cool; lips bluish; breathing short, hurried, and accompanied by a great sense of oppression; pulse small, frequent and feeble; and his expectoration bloody mucus. Percussion revealed almost entire dullness over the greater part of the right side of the chest, with a lesser degree of the same at the lower, lateral part of the left side. There was also strong vibration of voice, or bronchophony, over the right side, and tubular, respiratory sounds. These symptoms and physical signs indicated extensive and rapidly-increasing pneumonic exudation; so extensive, indeed, as to threaten speedily fatal results. Sinapisms were applied freely, both to the chest and extremities; and six grains of carbonate of ammonia dissolved in camphor-water, with the addition of a little camphorated tincture of opium, were given between each of the doses of the emulsion. He also took, during that night, a few times, small doses of brandy in sweetened water.

At my visit on the following day, which was yesterday, the surface and extremities had become warm, but the countenance was still depressed, and lips leaden-hue; the pulse soft and frequent; breathing short and imperfect; mind dull and drowsy; bowels loose; some cough, and expectoration composed of frothy mucus mixed with blood, with the physical signs the same as just described. The carbonate of ammonia mixture, alternately with the emulsion, was directed to be continued, with two grains of sulphate of quinine added to each dose of the latter, and a blister-plaster applied to the right side of the chest. To-day you find him still with a depressed, dull expression of countenance, pro-labia not quite so bluish, and the respiration a little more full; pulse still soft and weak, but less frequent; expectoration more free and less bloody, with nearly the same physical signs as yesterday. After the members of the class had examined the patient, it was stated that the principal objects of further treatment were to sustain the patient—more especially the tone of the vascular system—and to promote the re-absorption of the pulmonary exudations. As the looseness of the bowels had ceased, the emulsion of oil of turpentine and laudanum were omitted; but the two grains of quinine was continued, in connection with the carbonate of ammonia solution, every four hours, and a teaspoonful of the ordinary muriate of ammonia mixture given alternately with it. The regular administration of simple nourishment was also enjoined, as of equal importance with the giving of medicine.*

*Sub-Acute Hepatitis, with Dropsy.*—Mr. ——, aged 68 years, was admitted to the hospital about the first of October, 1873. He was seven feet and ten inches high; measured fifty-two inches around the chest; and when in good health weighed 395 pounds. For several years he had been on exhibition in different parts of the country as a noted giant. His health had been failing for several months, and when admitted to the hospital he had slight fever, dull pain in the right hypochondrium, loss of appetite, general weakness, and œdema of the lower extremities. On closer examination, there appeared to be a sub-acute inflammation of the liver; a weak and unsteady cardiac impulse, indicating fatty degeneration; moderate serous effusion into the cavity of the peritoneum, in addition to the œdema of the extremities. The urine was scanty, but not albuminous, and the bowels costive. The treatment adopted appeared to have no other effect than to palliate some of the more prominent symptoms. His strength continued to fail; a sense of oppression and dyspnœa was felt on assuming the recumbent posture, which steadily increased, until he was obliged to keep his chair day and night; and finally pericardial effusion with pneumonic congestion of the left lung hastened the fatal result. No post-morten was allowed.

*Acute Pleurisy.*—Mr. C——, a laboring man, native of Ireland, aged about 35 years, was admitted into the hospital four days since. The history of the case was given to the class, as follows: Two days before admission, or six days since, he was attacked with chilliness, acute pain in the sub-axillary region of the right side, shortness of breath, and some cough, followed by fever. At the time of his admission all these symptoms continued, with the addition of strongly marked dullness, on percussion, over the whole of the right side; absence of respiratory sounds, except at the apex and anterior upper margin

* This patient continued to improve slowly, until convalescence was established.

of that lung, and only very slight expansion of that side on inspiration: while the expansion and respiratory sounds of the left side of the chest were natural. The expectoration was scanty, and sometimes mixed with blood; the pulse 100 per minute, soft and regular; tongue coated; urine scanty; temperature of the body 102° F. Regarding the case as one of pleuritic inflammation, complicated with slight pneumonia and rapidly increasing serous effusion, a moderate-sized blister-plaster was applied to the lower part of the right side of the chest, and one teaspoonful of the following prescription given every three hours:

℞.—Hydrochlorate of ammonia, ʒ iij.

Tart. ant. and pot., grs. ij.

Sulph. morph., grs. iij.

Tinct. digitalis, ℥ i.

Syrup liquorice, ℥ iij.—Mix.

This treatment has been followed by a disappearance of all acute pain; a subsidence of the general febrile symptoms; and a disappearance of the blood from the expectoration. But, on direct individual examination, the members of the class still find complete dullness on percussion over the lower half of the right side of the chest; silence on auscultation over the same part; and only very slight expansile motion in inspiration. The infra-clavicular region of the right side was somewhat flattened, not quite as resonant as natural, and by auscultation gave a sub-crepitant rale in inspiration, and a prolongation of the respiratory murmur through the act of expiration. The physical condition of the left side appeared natural. In commenting on the case, attention was called to the dullness and silence of the axillary, sub-axillary, and posterior regions of the right side, as indicative of pleuritic effusion, while the depression beneath the clavicle, and prolonged respiratory murmur in the same region, justified a suspicion of tubercular deposit. In connection with this, attention was also called to the fact that the patient had a pale, cachectic look, was thin in flesh, and had been subject to frequent attacks of cough for more than a year. These facts, in connection with the present physical condition of the chest, render it very probable that the upper part of the right lung was studded with crude tubercular deposits before the recent inflammatory attack: a fact of much importance in its bearing upon both the prognosis and the treatment. A continuance of moderate counter-irritation on that side, with mild diuretic and alterative medicines internally, will, doubtless, soon remove what remains of the effects of the pleurisy. But if there are tubercular deposits, these will remain, and not only keep the patient from recovering good health, but will also present a strong tendency to take on softening, and the more active symptoms of consumption. This should be anticipated by putting the patient, as early as possible, on such remedies as will promote healthy, active nutrition, and such hygienic regulations as are known to counteract the progress of tuberculosis.

